# Esketamine for treatment resistant depression: a trick of smoke and mirrors?

**DOI:** 10.1017/S2045796019000751

**Published:** 2019-12-16

**Authors:** C. Gastaldon, D. Papola, G. Ostuzzi, C. Barbui

**Affiliations:** Department of Neuroscience, Biomedicine and Movement Sciences, Section of Psychiatry, World Health Organization Collaborating Centre for Research and Training in Mental Health and Service Evaluation, University of Verona, Verona, Italy

**Keywords:** Esketamine, evidence-based medicine, FDA, regulatory policies, treatment-resistant depression

## Abstract

In March 2019, the US Food and Drug Administration (FDA) approved a nasal spray formulation of esketamine for the treatment of resistant depression in adults. Esketamine is the S-enantiomer of ketamine, an FDA-approved anaesthetic, known to cause dissociation and, occasionally, hallucinations. While ketamine has not been approved for depression in the USA or in any other country, it has been used off-label in cases of severe depression. This commentary critically reviewed the evidence on esketamine submitted to the FDA, aiming to draw implications for clinical practice, research and regulatory science.

In March 2019, the US Food and Drug Administration (FDA) approved a nasal spray formulation of esketamine for the treatment of resistant depression in adults. Treatment-resistant depression (TRD) refers to a depressive episode with inadequate response to at least two antidepressant (AD) trials of adequate doses and duration. According to the FDA label, esketamine is indicated in TRD in association with AD treatment. This new drug has been under review by the European Medicine Agency (EMA) for approval and licensing, and received a positive feedback and may be soon available for clinical use also in European countries.

Esketamine is the S-enantiomer of ketamine, an FDA-approved anaesthetic. While ketamine has not been approved for depression in the USA or in any other country, it has been used off-label in cases of severe depression (Daly and Singh, 2018; Popova *et al*., 2019; Zhang and Hashimoto, 2019). However, ketamine is used for recreational purposes because it produces desired mental and behavioural changes, such as euphoria, and perceptual changes, such as dissociation and, occasionally, hallucinations (Caddy *et al*., 2015). These effects, together with a risk of abuse and misuse, made ketamine a widespread street drug, also known as ‘Special K’ (Zhu *et al*., 2016).

Against this background, in this commentary, the evidence on esketamine submitted to the FDA was reviewed, aiming to draw implications for clinical practice, research and regulatory science. The FDA website was searched using the term ‘esketamine’ (up to June 2019), and all documents were downloaded and independently inspected by two investigators. Phase III studies were identified and, using standard Cochrane meta-analytical methods (Higgins and Green, 2011), efficacy and acceptability data were extracted and re-analysed.

Critical inspection of the FDA documentation revealed a total of four phase III trials (Ochs-Ross *et. al.*, 2019; Daly *et al*., 2019; Fedgchin *et al*., 2019; Popova *et al*., 2019). Three were short-term placebo-controlled efficacy trials conducted in participants suffering from TRD. The main characteristics of the three efficacy studies are summarised in Table 1.

**Table 1.** Characteristics of the three short-term esketamine efficacy trials submitted to the FDA

Reference number	Participants	*N*	Treatment (N)	Treatment2 (N)	Control (N)	Duration (weeks)	Outcome
TRANSFORM 1 (NCT02417064)	Adults with TRD, age <65	342	Esketamine 56 mg + AD (115)	Esketamine 84 mg + AD (114)	Placebo + AD (113)	4	Change from baseline to day 28 at the MADRS
TRANSFORM 2 (NCT02418585)	Adults with TRD, age <65	223	Esketamine flexible dose + AD (114)	–	Placebo + AD (114)	4	Change from baseline to day 28 at MADRS
TRANSFORM 3 (NCT02422186)	Elderly with TRD, age >65	137	Esketamine flexible dose + AD (72)	–	Placebo + AD (65)	4	Change from baseline to day 28 at MADRS

MADRS, Montgomery–Asberg Depression Rating Scale; TRD, treatment-resistant depression; AD, antidepressant.

TRANSFORM-2 is the only short-term efficacy trial that found a significant difference between esketamine + AD and placebo + AD. This trial showed that participants receiving esketamine had a reduction of 21.4 points at the Montgomery–Asberg Depression Rating Scale (MADRS) (standard deviation (s.d.) 12.32) *v.* a reduction of 17.0 (s.d. 13.88) in the placebo group at day 28, with a mean difference between the two groups of 4.4 points. TRANSFORM-1 and TRANSFORM-3 failed to show a significant difference in terms of efficacy between esketamine + AD and placebo + AD.

The fourth study is a withdrawal placebo-controlled clinical trial (SUSTAIN-1, NCT02493868) (Daly *et al*., 2019). A total of 297 patients who achieved stable remission or stable response (without remission) with esketamine + AD were randomised to continue esketamine + AD (*N* = 152) or continue AD and switch esketamine to placebo nasal spray (*N* = 145). The primary outcome was time to relapse (Daly *et al*., 2019). Compared with AD + placebo, AD + esketamine decreased the risk of relapse by 51% (hazard ratio (HR) = 0.49; 95% confidence interval (CI) 0.29–0.84) in patients who had achieved stable remission, and 70% (HR = 0.30; 95% CI 0.16–0.55) in those who had achieved stable response.

Re-analysis of the primary efficacy data of the three phase III short-term studies (four comparisons in total, as for one three-arm trial each arm was considered separately *v.* placebo) revealed an overall mean difference of −4.08 (95% CI −6.20 to 1.97, *I*^2^ = 0%, three studies, 641 participants), suggesting that, in comparison with placebo, esketamine may improve depressive symptoms reducing by 4 points the MADRS score (Fig. 1). Of the four comparisons reported in Fig. 1, only one revealed a significant difference between esketamine and placebo.

**Fig. 1.**
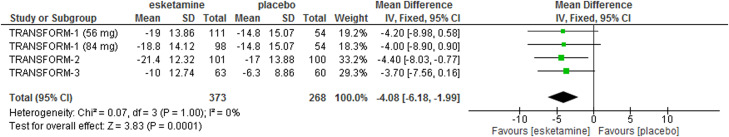
Mean difference between esketamine and placebo at day 28 (study endpoint) measured with the Montgomery–Asberg Depression Rating Scale (MADRS).

Pooling data on acceptability (dropouts due to any cause) showed that esketamine was significantly less acceptable than placebo (relative risk (RR) = 1.63, 95% CI 1.02–2.60, *I*^2^ = 0%, three studies, 711 participants). Re-analysis of data on the incidence of dissociation showed that esketamine increased by seven times the risk of this adverse effect over placebo, with approximately 25% of patients receiving esketamine who experienced severe dissociation during treatment (Figs 2 and 3).

**Fig. 2.**
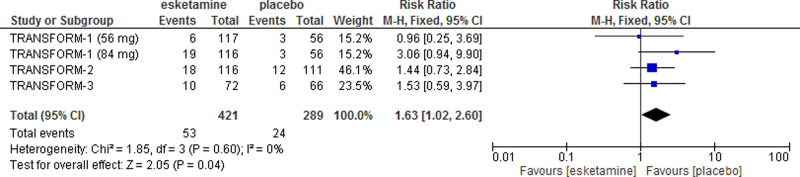
Acceptability of esketamine *v.* placebo at day 28 (study endpoint), measured as drop-outs due to any cause.

**Fig. 3.**
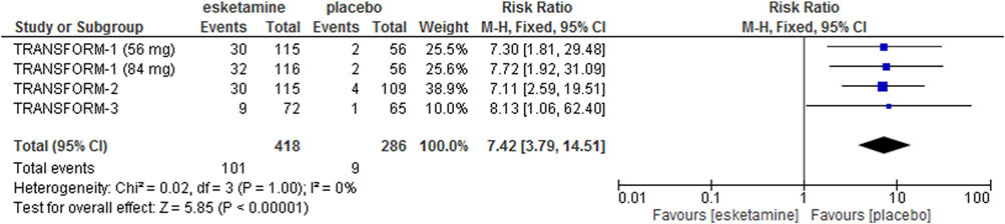
Risk of dissociation of esketamine *v.* placebo, measured as the proportion of patients experiencing this adverse effect.

Re-analysis of the clinical data on esketamine submitted to the FDA offered a unique opportunity to reflect on the evidence supporting the licensing of this new agent and, more generally, to critically appraise the approval process for new psychotropic drugs.

A first consideration refers to the efficacy of esketamine in TRD. Of three randomised trials submitted to the FDA, only TRANSFORM-2 was able to demonstrate the superiority of esketamine over placebo, while the other two trials showed similar mean differences in change scores without statistical significance. Re-analysis of the three trials revealed an average reduction of 4 points at the MADRS, a scale that scores from 0 to 60. Although this difference is statistically significant, its clinical meaning seems very uncertain. The authors of the three studies reported that a difference of at least 6.5 points at MADRS between esketamine and placebo should be observed to make a claim of clinical significance (Daly *et al*., 2019), based on the results of phase II studies (Daly *et al*., 2017). This implies that an average difference of 4 points may unlikely translate into a clinically meaningful beneficial effect under real-world circumstances, and additionally underlines a need for putting these results into a wider context, as they do not match with the author expectations. It could be argued that the reduction in the MADRS score from baseline to week 4 (−21.4 in the esketamine group *v*. −17.0) showed in the TRANSFORM-2 trial was clinically significant and higher as compared with other AD trials. However, it should be noted that this is not related to the esketamine effect itself, as a clinically important reduction in the MADRS score was detected also in the placebo group. It is, instead, most likely to be related to the high intensity of care received by patients in these trials, as compared to real-world practice.

A second consideration is that this difference was calculated against placebo, and not against an active comparator, such as, for example, a fixed-dose combination of olanzapine and fluoxetine, which is a licenced treatment in the USA for TRD. Interestingly, the FDA accepted studies that did not compare esketamine against the only available FDA-licensed gold-standard. Moreover, other treatment combinations, which might have been used as pragmatic control conditions, are often employed in clinical practice for TRD (Sanacora *et al*., 2017; Kim *et al*., 2019), but, according to the FDA, a demonstration of efficacy against placebo is enough to grant a marketing authorisation. A similar regulatory requirement is followed in Europe by the European Medicines Agency (EMA). We argue that this regulation is a real disservice as it allows the marketing of new drugs that may be less effective, or more harmful, than others already in use.

A third consideration is the extremely rapid onset of effect observed in TRANSFORM-2. One might wonder whether this effect means that esketamine was successful in rapidly correcting an underlying brain abnormality, which clinically was observed as a rapid improvement in depression scores or, rather, that esketamine just modified some brain processes that impacted the depression scores, as many psychoactive substances are able to induce. This possibility, known as a drug-centred model, suggests that drugs may produce a global state characterised by a range of physiological and psychological alterations. These alterations are likely to interact with the symptoms of mental disorders in ways that may sometimes be beneficial (Moncrieff, 2018). The rapid changes induced by esketamine make the drug-centred model particularly plausible.

For esketamine, understanding whether this rapid change in depression scores is due to an improvement of depression or just to a temporary effect of the drug on some brain mechanisms is of paramount relevance, as depression is a recurrent condition (NICE, 2018), and TRD is a particularly severe form of depression with symptoms persisting over long periods of time. It would be important to know if this acute effect is maintained in the long-term. For esketamine, however, long-term data are completely lacking. We know from SUSTAIN-1 that participants who discontinue esketamine after improvement with AD + esketamine are more likely to relapse in comparison with those who do not discontinue (Daly *et al*., 2019; Kim *et al*., 2019). However, it is well-known that this type of design tends to overemphasise the efficacy of maintenance treatment, as the comparison group is at extremely high risk of relapse, considering that treatment is abruptly stopped soon after improvement (Paykel, 2001; Pringsheim *et al*., 2016; NICE, 2018). Moreover, the high rate of rapid relapses observed after just 2 weeks from discontinuation might be interpreted as particularly worrisome, as it may suggest that the abnormal brain state induced by esketamine has caused rebound symptoms when suspended.

Additionally, the target population of withdrawal trials such as SUSTAIN-1 is different from the population recruited in efficacy short-term trials, where participants suffering from acute depression are recruited. As a consequence, generalizing results from a withdrawal trial to patients with a current depressive episode is difficult (Guyatt *et al*., 2008; Post *et al*., 2013), and extrapolating efficacy in the long-term seems very problematic. Methodologically, this is the first time that the FDA Division of Psychiatry Products considered a randomised withdrawal trial as one of the two adequate and well-controlled trials comprising substantial evidence of effectiveness, as noted by Kim *et al.* in a recent paper (Kim *et al*., 2019).

Fourth, although the FDA emphasised that the safety of esketamine was the main concern given the well-known risks associated with ketamine (FDA, 2019), studies showing that esketamine is less risky than ketamine are lacking. Before approval, the FDA committee wrote in its documentation that ‘data on safety of ketamine may be considered relevant to discussions regarding the safety of esketamine. […] The risks of abuse and associated harms are important considerations in determining appropriate risk mitigation strategies and post marketing surveillance for esketamine, if approved’ (FDA, 2019). Consequently, after esketamine approval, the FDA determined that a Risk Evaluation and Mitigation Strategy (REMS) (a drug safety programme required medications with serious safety concerns) was needed to help ensure that the benefits of the drug outweigh its risks. We argue that this implies approval without knowledge of the potential negative consequences of esketamine prescribing.

What can be extrapolated from the data submitted to the FDA, however, is that esketamine showed a significant worse acceptability profile as compared to placebo, with a higher proportion of participants dropping-out. This performance seems different than that of ketamine, as a recent Cochrane review found no difference between ketamine and placebo in terms of acceptability (odds ratio (OR) 1.90, 95% CI 0.43–8.47; *I*^2^ = 24%; five studies, 139 participants) (Caddy *et al*., 2015). Indirectly, these results pose some concerns on the overall acceptability of esketamine, suggesting that it may be even worse than ketamine.

Additionally, the FDA reported that the safety concerns for esketamine, for which a REMS was planned, are misuse, abuse, dissociation and sedation (FDA, 2019; Kim *et al*., 2019), which are known adverse effects of ketamine. During the trials, no misuse or abuse was observed, but this was due to the fact that esketamine was administered under strict medical supervision (at least 2 h), and only in highly specialised centres. For dissociation, re-analysis of the three efficacy trials revealed that the risk of this adverse event was almost seven times higher in the esketamine group as compared with placebo, with approximately 25% of patients experiencing severe dissociation during acute treatment with esketamine. Again, we argue that further evidence on safety is urgently needed, given these preliminary signs suggesting that esketamine may not be safer than ketamine.

The example of esketamine shows that current rules governing the registration of new psychotropic drugs are based on the concept of absolute efficacy, which implies that a difference against placebo, and not against an active comparator, makes a new investigational product eligible for registration. We suggest that the concept of absolute efficacy should be replaced by the concept of added value, which implies that evidence from studies comparing an investigational product with an active comparator should guide the drug approval process. We have additionally proposed that the evaluation process of psychotropic medicines should be complemented with regulatory meta-analyses of all relevant clinical studies to define their efficacy and tolerability profile (Barbui *et al*., 2017). Based on the results of regulatory meta-analyses, regulatory authorities may develop a more systematic approach to summarise the beneficial and harmful effects of new psychotropic drugs. Also, for esketamine, a number of other studies could have been evaluated, and the FDA itself reported that in other studies, six suicides happened and all of them were in the esketamine arm (FDA, 2019; Schatzberg, 2019). Excluding these data from the approval process could lead to misleading conclusions on safety, as recently pointed out by Schatzberg (2019).

Although the important limitations of the evidence base, esketamine was labelled as a breakthrough therapy for TRD. The strategy to approve it as REMS could help addressing some safety issues, but this will require a long time and exposure of many persons with depression to this new agent. Considering the explanatory nature of existing studies, large pragmatic trials are urgently needed to better define the place in therapy of esketamine, aiming to clarify if there is more than just smoke and mirrors.

Finally, we argue that the EMA should take into due account all these critical issues when assessing the marketing authorisation of esketamine for Europe, and, more broadly, we call for a radical change of current regulatory rules for psychotropic drug approval.
